# Physical Activity as Part of an Intramural Health Promotion Programme for People with and without Chronic Diseases. A New Tool in Health Care Run by a Public Social Health Insurance

**DOI:** 10.3390/ijerph17207491

**Published:** 2020-10-15

**Authors:** Thomas E. Dorner, Gudrun Wolner-Strohmeyer, Christian Katzenbeisser, Christian Lackinger, K. Viktoria Stein

**Affiliations:** 1 Social Insurance Fund for Public Service, Railway and Mining Industries, 1080 Vienna, Austria; gudrun.wolner-strohmeyer@bvaeb.at (G.W.-S.); Christian.Katzenbeisser@bvaeb.sv.at (C.K.); 2 Karl-Landsteiner Institute for Health Promotion Research, 3454 Sitzenberg-Reidling, Austria; inminister@gmx.at (C.L.); viktoriastein@yahoo.co.uk (K.V.S.)

**Keywords:** sustainable health promotion, integrated care, healthy adults, chronic conditions

## Abstract

Background: Regular physical activity is a corner stone for healthy living, and preventing the onset or progression of diseases. The Social Insurance Fund for Public Service, Railway and Mining Industries is building an intramural health promotion facility in Austria with the aim to provide a comprehensive evidence-based health promotion programme for their insured. The target group are all people who, regardless of their health status and the presence of diseases, are ready to make their lifestyle more health-oriented. The health promotion facility offers health promotion measures in five areas: promoting physical training, optimizing nutritional patterns, managing everyday stress, increasing social capital, and improving health literacy. The focus is on increasing resources and on overcoming barriers. Depending on age, previous illnesses, range of motion, stress level, body weight and personal aims and expectations, the measures are individually tailored. The stay is divided into a two-week initial stay and a follow-up week. A comprehensive scientific evaluation concept of all measures and the entire stay is an integral part of the design. Conclusion: This project combines the advantages of comprehensive active health promotion, and an intramural stay. It is a pioneering social insurance project for sustainable health promotion and integrated care.

## 1. Introduction

In the health and social system, new ways of promoting physical activity in people with or without chronic diseases are inevitable so that as many people as possible can achieve the highest possible level of health. The pioneering non-profit project of the statutory public social insurance described below offers a possibility of how physical activity, and other lifestyle-based health promotion measures, can sustainably be implemented. This new intramural health promotion stay will be granted by the social insurance at the request of the insured and after medical justification. During the stay, the insured are on sick leave and the social insurance fund covers almost the entire costs. Since this is a pioneer project, all applied measures are going to be subject of comprehensive scientific evaluation.

### 1.1. Physical Activity

Regular physical activity is a corner stone for healthy living. Generally, physical activity covers every bodily movement that leads to an increase in energy expenditure. Thus, physical activity includes light, moderate and vigorous intense activities [1]. Physical training aims to improve physical function and morphological changes, includes endurance and strength training and is usually performed with moderate to vigorous intensity.

According to current international physical activity guidelines adult people, including healthy individuals and those with chronic conditions and diseases should be physically active on a regular basis. Above all, the change from “physically inactive” to “a little physically active” is an important first step. To increase health, all adults should perform at least 150 min to 300 min per week of endurance-oriented physical activity with moderate intensity or 75 min to 150 min per week of endurance-oriented physical activity with vigorous intensity or perform an appropriate combination of endurance-oriented physical activity with medium and higher intensity. Additionally, all adults should perform muscle-strengthening exercises on two or more days of the week, taking into account all large muscle groups [2,3].

Although the physical activity guidelines are very well-known in the public [4], according to self-reported population-based data, more than half of the adult Austrian population do not fulfill the endurance-oriented recommendations and more than two thirds do not fulfill the recommendations for muscle strengthening [5]. In patients with chronic medical diseases, the situation is even worse. In order to improve the adherence to physical activity recommendations especially in patients with chronic diseases, low threshold connections between the health care systems, where patients with chronic diseases are very frequent, and physical training facilities, where these patients are very rare, are required. However, the implementation of physical training as a health promotion tool in the medical care system faces many obstacles. There are several reasons for this: first of all, physical training often needs to be promoted among older persons who are historically unfamiliar with the principles of training and don’t experience physical activity as a joyful activity. Additionally, there is often an adherence problem. As most people know from personal experience, it is easier to initiate physical training, but it is difficult to keep up a routine in the long run. Furthermore, and this is probably the biggest obstacle, there is a lack of infrastructure and qualified instructors to implement and maintain physical training in people with chronic diseases. In many countries, local sports clubs are traditionally associated with athlete’s sports and are not involved in leisure, recreational or health-related sports programmes [6]. Additionally, local sports facilities like sports clubs or commercial fitness centres often lack evidence-based, standardised programmes and don’t have a link with the health care setting [7]. Therefore, new structures are required in which the health needs of people with chronic conditions and diseases, risk factors, and healthy people who want to improve their health are taken into account, providing evidence-based programmes, tailored to the health needs of the participants, and supported by well-established links between the medical care providers, sports facilities and other services supporting lifestyle changes. 

### 1.2. Physical Training, Health, and Chronic Diseases

Physical training is a systematic process that includes at least moderate intense endurance or strength training. Physical training is among the most established methods for sustainable health promotion and elevation of well-being, independent from the health status of a person. Additionally, physical training is important in the management of certain chronic conditions and diseases. These include cardio-metabolic risk factors, overweight and obesity, rheumatologic diseases, cancer, musculo-skeletal disorders like osteoarthritis, osteoporosis, and chronic back pain, hypertension, other cardio-vascular diseases, dyslipidaemia, type 2 diabetes mellitus, multiple sclerosis, and common mental disorders like stress-related disorders, anxiety or depression. In many chronic medical conditions, physical training has a multitude of beneficial functions: (1) physical training increases well-being and quality of life, irrespective of the possible positive impact on the underlying condition; (2) physical training is part of the treatment of the disease, especially in persons where lack of physical activity is part of the pathogenesis of the disease; (3) physical training can postpone the progression of the disease; (4) physical training can prevent the development of co-morbidities, which could trigger adverse outcomes; (5) physical training can improve functionality and relieve symptoms (such as pain) and can thus help to better cope with the activities of daily living [2]. 

For social insurance funds, and their branches health insurance, accident insurance, and pension insurance, physical activity and physical training are important preventive tools. As physical training contributes to the prevention of diseases, it may thus reduce the amount of sick leave benefits and disease treatment expenses. Additionally, physical training contributes towards the prevention of falls and other accidents and therefore reduces the risk for accident-related treatment, rehabilitation and retirement due to invalidity. Furthermore, physical training enhances capacity to work, and therefore may prevent early or permanent exit from the labour market, thus again reducing the risks for disability pension or unemployment. 

### 1.3. Social Insurance System in Austria

In Austria, there are at present five social insurance funds, responsible for health, pensions, and accident insurance. Austrian citizens are assigned to a certain insurance fund by law according to their profession. One cannot choose the insurance provider oneself. The Social Insurance Fund for Public Service, Railway and Mining Industries, for instance, covers social insurance amongst others for civil servants and contract staff in public service (e.g., policemen/policewomen, military personnel, teachers, judicial staff, …), politicians, university staff, railway workers, miners, and their own employees, in total for more than one million Austrians (approximately 11% of all insured persons in Austria). By law, the social health insurance coverage protects individuals from risks of illness, inability to work, covers the cost of sickness treatment, medical care, rehabilitation, travel and transportation, sickness benefits and rehabilitation allowance, and it provides maternity benefits and maternity care. These tasks are usually carried out by external health providers who are under contract with the social insurance system. In addition, however, social insurance funds sometimes run their own facilities, such as hospitals, outpatient clinics, rehabilitation centres and spa facilities [8]. Health promotion and prevention are not established fields of activities in Austrian health insurance funds.

In addition to the offers of public social insurance funds, the health and wellness market has been a growing market segment for many years in Austria, and innumerous offers are available for health and wellness stays in spa hotels and similar facilities. While these wellness facilities and programmes may also have a health benefit, that is not their primary purpose and they cannot be regarded on an equal footing as a multi-disciplinary, evidence-based and comprehensive preventive programme, which has as its primary objectives to reduce risk factors, and increase health literacy, self-management and self-efficacy of the participants.

## 2. A New Intramural Facility for Health Promotion by a Public Social Insurance

The legal basis for the new intramural facility for health promotion is the law for stationary spa therapy (“Kur”) and rehabilitation. The traditional spa therapy was established more than two centuries ago in the Habsburg-monarchy and it is usually run and paid for by the public mandatory social insurances. The treatments are mostly passive such as balneologic, physical, or recreational methods and lectures. By law, the public social insurance can grant such a spa therapy, based on an application by the patient and a medical justification by a physician. The social insurance fund then evaluates the necessity for spa therapy or rehabilitation and grants or rejects the application [8]. The insured are allowed to apply for two stays in five years, with at least 18 months in between the two stays. The in-patient spa therapy is almost fully covered by the social insurance, with a small out-of-pocket payment per day, which is also regulated by law and graded according to the income of the insurees (currently between €0 and a maximum of €20.9 per day) [9]. Although the evidence on the effectiveness of spa therapy in Austria is limited, there are some proven positive effects of three-week spa stays on health. For example, spa therapy affects stress and the risk of burnout [10], has effects on reducing pain [11], and on lowering blood pressure [12]. 

In Austria, there are currently 83 facilities for in-patient rehabilitation. Additionally, there are more than 900 outpatient clinics, which are also classified as hospitals according to the Federal Hospital and Health Resort Act, and some of these also provide spa therapy [8]. The public social insurance funds in Austria own in total 41 facilities, providing in-patient rehabilitation or spa therapy [9,13,14,15,16]. Currently the Social Insurance Fund for Public Service, Railway and Mining Industries owns and manages nine facilities for medical rehabilitation or spa therapy for various medical indications [9]. A tenth facility with a focus on intramural health promotion for people with and without chronic diseases is currently under construction. In addition to these facilities, the Social Insurance Fund for Public Service, Railway and Mining Industries has contracts with 47 other facilities which provide in-patient rehabilitation or spa therapy, and they are owned by other social insurance funds or private partners, and they can be utilized by the insurees of the insurance fund [9]. In 2018, the Social Insurance Fund for Public Service, Railway and Mining Industries granted 13,527 in-patient rehabilitation applications and 17,925 in-patient spa therapy applications for their approximately 1 million insurees [17,18].

Based on the existing evidence, the traditions, and the laws regarding spa therapy, the Social Insurance Fund for Public Service, Railway and Mining Industries has decided to build a new facility with a focus on health promotion. The new health promotion facility is planned for launch in autumn 2021, with a capacity of 120 beds and 2200 participants per annum. This intramural health promotion facility is equipped with a 170 square meter auditorium, seminar rooms, a standard sports hall, a 25-metre swimming pool, a gym with endurance and strength devices, gym rooms for group training, a teaching kitchen, an outdoor motor skills park and an outdoor athletic ground, studios for creativity, and rooms for massage and hydrojet water pressure massages. In contrast to the existing spa therapy, the new facility will ask participants to take a more active role and make shared decisions with the health professionals regarding health targets, treatments and therapies. And, most importantly, all offers must be based on scientific evidence. In the case of lack of evidence, the measures are to be scientifically evaluated at the facility. A summary of the main differences between this new health promotion facility, a traditional spa therapy centre and rehabilitation is depicted in Table 1.

The health promotion stay covers three weeks in total. These are split into two weeks of initial stay and one follow-up week. At the end of the initial two weeks, patients and medical doctors agree on individual and specific lifestyle goals, based on the comprehensive evaluation of an interdisciplinary team of health professionals and according to the needs and targets of the participant. These individual goals should be SMART (specific, measurable, attractive, realistic, time-bound). Over the course of the following three months the participants are encouraged to implement the newly acquired skills in their daily routine and aim to reach their individual targets. Thereafter, the targets are evaluated in the follow-up week, possible obstacles to achieving the targets are identified and strategies are developed together with the participants to overcome those obstacles going forward. If necessary additional health resources are made available. 

The intramural health promotion stay offers activities in five domains:

- Promoting physical training
- Optimizing nutritional patterns
- Managing everyday stress
- Increasing social capital
- Improving health literacy

At the end of the follow-up week, SMART goals are again defined together with the participants. The time to reach these goals will be individually agreed and the participants are invited to evaluate these goals independently.

### 2.1. Target Groups and Basic Concept for the Health Promotion Programme

The target population for the intramural health promotion programme are adults who may be expected to profit from lifestyle improvements in terms of health status, and who are seriously prepared to change their health behaviour. The programme specifically focuses on persons of employment age. Among those, people with risk factors or non-communicable diseases, especially those linked to lifestyle factors are the main target group. However, the pre-existence of chronic diseases is not a prerequisite to participate. If there are pre-existing conditions, they need to be stable and not require intensive medical or nursing care. Exclusion criteria are mainly a lack of motivation, patients with decompensated diseases, and with acute infections.

In accordance with the procedure for in-patient rehabilitation or spa therapy, the possible participant has to apply for the intramural health promotion stay and a medical doctor has to justify the medical need for lifestyle modification. The chief medical service within the social insurance then accepts or declines this application, or makes suggestions for other options, i.e., medical rehabilitation or spa therapy, if these are more appropriate. The decision of the chief medical service is based on objective criteria, according to the medical history and the justification by the medical doctor. 

### 2.2. General Aims and Different Modules in the Intramural Programme

**Figure 1:**
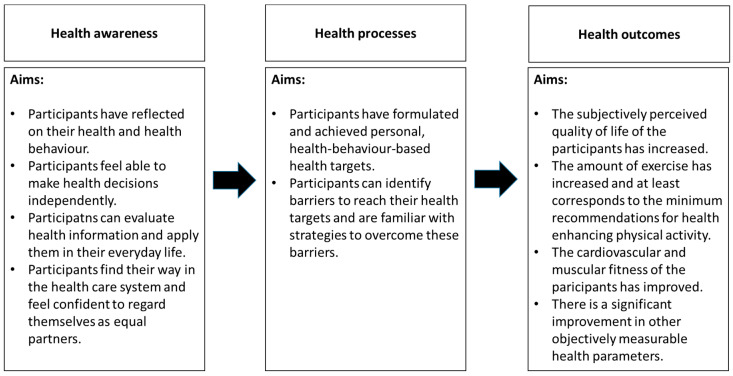
Health aims in different dimensions.

The general aim of the intramural health promotion programme is to improve the health of participants sustainably. To achieve this overarching goal, intermediary aims have been articulated in three dimensions: health awareness, health processes, and health status, (see Figure 1). 

### 2.3. Diagnostic Tools

In order to generate tailored health promotion measures, but also to evaluate the effectiveness of the measures, a comprehensive set of diagnostic tools will be implemented (Table 2). The set comprises of a general medical and physical examination, anthropometric parameters (height, weight, and waist circumference), bioimpedance analysis, and resting ECG, and in some cases 24-h ambulatory blood pressure or spirometry. Additionally, it includes evaluation of physical activity habits, activity tracking during the intramural stay, and physical fitness (muscle strength and endurance), along with diagnostic tools for nutrition, stress, social capital and health literacy. In advance of the initial stay in the health promotion facility, participants are asked to fill in a questionnaire with their medical history, their physical activity behaviour, their stress level, body weight and body height, and their personal expectations. This will be repeated before the follow-up week. All other diagnostic tools are performed in the first two days of the basic stay and repeated during the follow-up week. Additionally, laboratory parameters will be collected. These include a total and differential blood count, serum lipids, glycose, insulin, glycosylated haemoglobin, uric acid, parameters of chronic inflammation (interleukine-6, tumor necrosis factor, high sensitive C-reactive protein), diurnal cortisol profile and the body microbiome.

#### 2.3.1. Physical Fitness

The concentric one repetition maximum (1-RPM) is the gold standard to determine muscle strength and is evaluated within three to six attempts and a rest period minimum of 3 min between each repetition [19]. It represents the maximum, voluntary lifted weight for a specific strength exercise. It can be measured with a high level of safety with free weights, but it is rarely done in health care settings, because of the time needed [20].

Electronically controlled strength testing machines allow to determine the 1-RPM with one attempt. In contrast to isokinetic machines, electronically controlled concentric machines are not restricted to a constant velocity when a weight is lifted [21]. A microcontroller adopts the speed of movement comparative to lifting a free weight. Using such devices, the 1-RPM for seated leg press, chest press and seated rowing is determined within one single attempt at the beginning of the initial stay and will be repeated in the follow-up week.

Before muscle strength is assessed, participants will perform a standardized warm up procedure using a bicycle ergometer (at 70% of their estimated maximum heart rate). Then, the participants are scanned with a video system, so that individual positions and settings for the different strength testing machines can be pre-set automatically. 

To get familiar with the first strength testing machine and to verify the automatically adjusted position, participants are asked to perform 10 submaximal repetitions (at approximately 40% of the expected individual 1-RPM). After a short break, participants are instructed to perform a maximum repetition to determine the 1-RPM. After a minute of rest, participants are encouraged to do 10 repetitions continuously with a weight representing 70% of 1-RPM to measure muscular endurance [22]. Here the velocity during the concentric phase should be as high as possible. The devices do not only evaluate the weight: registering the velocity during each single repetition allows to calculate muscular power. The monitoring of the movement velocity is an additional parameter to quantify the load during strength training and testing [23]. This is the procedure for all three strength testing machines for big muscle groups. For the testing in the follow-up week, the same weight load as in the baseline tests is used to determine strength endurance. 

Additionally, the maximum hand grip strength is measured with a handgrip dynamometer [24,25]. The maximum isometric strength is measured during the hand force test according to a standardized procedure: the person sits, has adducted the shoulder and is in a neutral rotational position, the elbow in 90° flexion, the forearm in neutral position and the wrist between 0 and 30° dorsiflexion. The strength is measured twice on each side with a one-minute pause. The highest value is used for the evaluation [26].

The strength endurance of the lower extremity is measured using the 30-multiple sit-to-stand test (MSTS). The MSTS test measures the strength of the lower extremities by repeatedly standing up and sitting down on a chair in 30 seconds only with the help of the legs. The number of complete repetitions is noted [27,28].

Aerobic capacity is measured with an exercise stress test on a bicycle ergometer with electrical brakes. The exercise capacity will be evaluated in line with international and national guidelines for exercise testing [29,30,31]. Thus, the protocol for exercise tests includes an initial warm-up period at constant workload, following a progressive increase of the workload and finally a recovery period. According to the expected maximum performance, the increments are defined, so that exhaustion should be reached within 9–12 min and covers a minimum of 6 different levels [32]. Important outcome parameters in this examination are the endurance capacity measured in watts at maximum exhaustion and the watts per kilogram of body weight. Age and sex specific reference values are used to interpret the results. The exercise stress test will be done twice: first at the beginning of the initial stay and repeated during the follow up week. The exercise test is continuously ECG and blood pressure monitored, capillary lactate levels are taken, and, where appropriate, ventilation gases are obtained. The bicycle ergometer is performed to fulfil three aims: (1) to detect unknown cardio-vascular and respiratory diseases, (2) to exactly evaluate endurance fitness and trends over time, and (3) to measure respiratory and metabolic thresholds in order to create individual heart rate monitored training plans.

#### 2.3.2. Physical Activity Behaviour

Physical activity is measured with the European Health Interview Survey-Physical Activity Questionnaire (EHIS-PAQ) [33]. In this questionnaire, the extent of physical activity is recorded with seven questions, in three different activity domains. The amount of physical activity at the workplace, for transportation purposes (cycling and walking) and leisure-time physical activity in a typical week is assessed. In addition, the time for muscle strengthening physical activity in a typical week is recorded. The questions are analysed according to a standardized protocol [33]. 

During the stay in the health promotion facility, participants are equipped with fitness trackers [34]. The aim of this monitoring is to coach the participants in their perception of the amount, duration, and intensity of physical activity. Thus, sedentary (lying, sitting, standing) light-, moderate-, and vigorous intense physical activity as well as exercise sessions will be registered. Additionally, with wearables (handed out during the stay) the expended energy is monitored, but also sleep quality, and heart rate variability.

#### 2.3.3. Other Diagnostic Tools

Questionnaires applied at the health promotion facility are a stress questionnaire (e.g., PSQ) [35], one regarding health resources (Rutz Human Condition 4—Satisfaction Index) [36], one on quality of life (WHO-QOL-Bref) [37], a questionnaire on nutritional behaviour (24-h recall) [38], on social capital (e.g., OECD questionnaire on social capital) [39,40], and one on health literacy (e.g., HLS12) [41]. 

### 2.4. Health Promotion Interventions

In the intramural health promotion facility, three different types of modules are offered: The basic modules are the same for all participants and are completed during the initial stay. The core modules are tailored to the special health needs and requirements of the participants and are completed in a closed group of 10 persons during the initial stay as well as in the follow-up week. This group will form a community of peers, which should offer support and motivation beyond the programme. The elective modules are freely selected by the participants and are also taken during the initial stay and in the follow-up week. This amounts to a total of at least 45.5 h of activities (2730 min), spread over 3 (2 + 1) weeks. They are divided into 10 h of basic modules, 26.5 h of core modules and at least nine hours of elective modules. In addition to these guided sessions, additional activities can be undertaken independently (e.g., independent use of the fitness area, the swimming pool, the motor skills park or the hydrojet water pressure massages) (Table 3).

The basic module is mandatory for all participants. The welcome lecture takes place on arrival days for all new participants. The remaining lectures can be held in large groups with up to 80 people. In terms of content, they do not build on each other and must be completed during the initial stay (therefore basic module). The basic module consists of the following lectures:

- “Welcome, introduction, concept” (60 min)
- “Physical exercise and health and basics of training principles” (90 min)
- “Basics of a healthy diet” (90 min)
- “Social capital and health” (90 min)
- “What is health literacy?” (90 min)
- “Dealing with everyday requirements—stress management” (90 min)
- “Methods of resilience—How can I strengthen resilience?” (90 min)

Based on the results of the screening questionnaire before the initial stay (extent of physical exercise, stress level, and body mass index), and depending on their age and medical history, but most importantly based on the personal preferences, participants are assigned to a core module. It is intended to form homogeneous groups of 10 people with similar health needs prior to arrival. These groups are then scheduled to arrive on the same day and remain together for the entirety of the programme.

Core modules consist of:

- 8 h of physical training (fitness area, gym, swimming pool, aqua gym, outdoor facilities)
- 6 h teaching kitchen (2 units of three hours each)
- 2 h nutrition seminar
- 5 h of psychology (behaviour change, conversation therapy, time management, stress coping, relaxation techniques, mindfulness exercises, biofeedback) divided into two units of 60 min and two units of 90 min.
- 3 h seminar on social capital (2 units of 90 min each)
- 90 min seminar on health literacy
- 1 h of massage

The composition of the core modules is identical for all groups; however, they differ in their contents (e.g. in terms of physical activity, aqua gymnastics for weight reduction, recreational training for stress reduction, intensity of training adapted to the activity level of the group, etc.).

The elective modules are chosen in consultation with the multi-professional team when planning the individual programme as part of the first medical consultation. Saturday morning is dedicated to the elective modules (three hours on three Saturdays). In addition, extra massages, hydrojet water pressure massages, as well as independent use of the fitness area (endurance or strength), the swimming pool and the outdoor motor skills park can be taken advantage of as part of the elective modules. At least one creative workshop is mandatory. In the case of certain medical diseases, tailored lectures are recommended (e.g., lifestyle and hypertension, physical activity in the management of type 2 diabetes mellitus, etc.).

### 2.5. Novelty of This Intramural Health Care Facility within the Health Care System

Health promotion and prevention are typically classed as public health activities and are thus associated with primary and community care, ultimately lying in the responsibility of the individual. With the rise of chronic and lifestyle-related diseases, education and support around nutrition, physical activity and mental health have become even more relevant, but the onus still remains largely on the individual. There is a plethora of examples and evidence available on patient education, health promotive and preventive measures in the context of disease management programmes and integrated care models as a consequence of the realisation that it is far more effective to avoid chronic conditions or the progression and exacerbation thereof, than targeting the top 20% of patients with multi-morbidities [42,43,44]. Typically, these programmes offer nutritional guidance, recommendations on physical activity and self-management support. Some go so far as to collaborate with gyms or sports facilities or offer psychological support. Innovative approaches include Halton CCG in England, which started as a collaborative between primary health care, the municipality and the local rugby club to tackle health inequalities, social isolation and multimorbidity by using the draw of the rugby team to educate and motivate community members to improve their health [45]. Integrated health systems such as Canterbury, New Zealand [46], NUKA System, Alaska [47], or Healthy Kinzigtal, Germany [48] offer a whole range of activities for their local populations to promote health and wellbeing and prevent chronic diseases. However, to the best knowledge of the authors, there are no programmes which offer a holistic, prolonged and long-term health promotion and prevention programme, combining intramural stays with self-management and targeting a population of working age with the explicit goal to support health and wellbeing, and address the pertinent risk factors for chronic conditions and mental health issues collectively. A further innovative aspect is the evaluation and monitoring framework, which is an integral part of the programme. While calls to do so abound in the literature, in practice high quality evaluation is still the exception rather than the rule [49,50].

### 2.6. Considerations Regarding Cost-Effektiveness

The costs for this programme are estimated to be between €2000 and €3000 per person per stay, dependent on which cost items are included in the calculations, i.e., only costs for personnel, materials and consumables, or also depreciation costs for the building or administration costs for the central office of the social insurance institution. €3000 multiplied by a maximum of 2200 participants per year leads to annual expenses of an estimated €6.6 million. These costs are almost completely covered by the public social insurance (apart from a small out-of-pocket payment for the participants). In contrast, successful health promotion initiatives save social security expenses. These savings include expenses for medical treatment, pharmaceuticals and medical devices in the extramural health care setting, costs for medical treatment in hospitals, costs for sick leave, and costs for health-related early exit from the labour market, i.e., disability pensions and unemployment benefits. According to an American calculation, 2.4 to 5% of the total health care expenditure is attributable to lack of physical exercise [51]. An Austrian calculation estimates that approximately 3.6 to 5.5% of the total health care expenditure is attributable to physical inactivity [52]. The health expenditures for health care of the Social Insurance Fund for Public Service, Railway and Mining Industries are approximately 2.6 billion Euro per year [53]. Using these estimates of 2.4 to 5.5% and applying them to the Austrian context, the Social Insurance Fund for Public Service, Railway and Mining Industries incurs an estimated €62.4 to 130 million in health expenditure each year, which is attributable to lack of physical activity. Additionally, the public pension fund and unemployment insurance accumulate potentially preventable costs due to disease-related early exit from the labour market. Thus, it is to be expected that the costs per person spent in the new health promotion facility described in this article will be offset by savings along the life course of the insurees through reduced risk of chronic disease and early retirement, as well as higher productivity. However, no solid data are yet available for Austria to be able to calculate the true cost-effectiveness of programmes like this, which is why one of the objectives of the facility is also to evaluate the interventions for their cost-effectiveness and establish an evidence base. 

## 3. Outlook and Conclusions

### 3.1. Further Development

The times between the initial and the follow-up stay, and especially after the follow-up stay are crucial for sustainability of the newly acquired lifestyle measures and for long-term adherence. Modern electronic self-management tools like online tools or mobile apps can be used to enable long-term support through health professionals and enable interaction with and motivation among the peer group of 10 created during the stay [54,55]. Such tools are planned to be developed and introduced. Additionally, the connection with other health promotion, prevention, health care programmes, and facilities of the Social Insurance Fund for Public Service, Railway and Mining Industries, as well as links to stakeholders beyond the classic health care and social insurance setting like sports facilities, social sector, non-profit organisations for voluntary work, etc. need to be established.

The cooperation with primary care and with general practitioners will be sought especially, since primary care physicians play a key role in health promotion and in supporting a healthy lifestyle [56,57,58].

Furthermore, a comprehensive scientific evaluation of all parts in this intramural health promotion facility is planned. This includes not only evaluation regarding effectiveness of measures taken during the stay, and adherence of the participants between the initial and the follow-up stay, and after the follow-up stay, but also the long-term effectiveness of the program. For this, prospectively, social insurance routine data of the participants, like medication prescription, number, duration, and diagnoses for sickness absences, number, duration and diagnoses for hospital treatment, risk and reason for disability pension, but also risk for nursing care needs, and even mortality risk can be taken and compared to matched individuals who did not participate in the intramural health promotion programme. 

### 3.2. Conclusions

This form of intramural health promotion facility offers a unique opportunity to realise an integrated, person-centred and long-term approach to health promotion and disease prevention by combining elements of intramural, primary and community-based care with supported self-management. By following a holistic understanding of health and wellbeing the facility will be able to truly offer a bio-psycho-social approach tailored to the needs of the individual. Long-term sustainability and adherence will be supported by connecting the participants with services in their neighbourhood and offering online self-management support as well as creating a community of peers beyond the intramural stay. The comprehensive evaluation and monitoring framework will enable the establishment of a continuous learning cycle to improve services and outcomes. As such this facility constitutes a novel and innovative approach to health promotion and prevention garnering the advantages of intramural, out-patient and community-based care, and connecting people to the resources, tools and services necessary to achieve the health and wellbeing targets important to them.

## Figures and Tables

**Table 1:** Similarities and differences between traditional rehabilitation, traditional spa therapy and the planned intramural health promotion facility.

Types of Intramural Facilities (Horizontal)Characteristics (Vertical)	Rehabilitation	Traditional in-Patient Spa Therapy	New Intramural Health Promotion Facility
Financial coverage	Public social insurance	Public social insurance	Public social insurance
Duration	3–6 weeks	3 weeks	3 weeks, split in 2 + 1 week
Focus	Indication-centred	Indication-centred	Person-centred
Time in the course of disease	After acute disease treatment	After acute disease treatment	Independent from existence of diseases
Health model	Bio-psycho-social (with emphasis on medical health)	Bio-psycho-social (with emphasis on medical health)	Bio-psycho-social (all dimensions equal)
Aims	Return-to-work; occupational and social rehabilitation; prevention of early exit from the labour market; prevention of reoccurrence or exacerbation of the disease	Prevention of reoccurrence or exacerbation of the disease; maintenance of ability to work	Gain in health in all dimensions; higher quality of life; improvement of ability to work
Orientation of measures	Active and passive	Mainly passive	Mainly active
Extent of measures	Variable	1400 min	2700 min
Screening before the stay	No	No	Yes
Diagnostics	Sufficient in connection with the disease	Basic	Comprehensive
Orientation on individual health goals	Partly	No	Yes

**Table 2:** Overview of the set of diagnostic tools.

Area	Measurement	Time of Measurement		
		Before the Initial Stay	Initial Stay	Follow Up Week
General medical and physical examination	Medical history	√		
	Medical anamnesis		√	√
	Lifestyle anamnesis		√	√
	Anthropometric parameters		√	√
	Bioimpedance analysis		√	√
	Resting ECG		√	√
	Laboratory parameters		√	√
	Sleep quality		√	√
Physical activity	Physical activity behaviour	√		
	Activity tracking		√	√
Physical fitness	Maximum muscle strength (1-RPM)		√	√
	Exercise stress test		√	√
	Submaximal muscle strength (70% of 1-RPM)		√	√
	Handgrip strength		√	√
	Strength endurance		√	√
Additional diagnostic tools for:	Nutrition		√	√
	Perceived stress	√		√
	Social capital		√	√
	Health literacy		√	√
	Sleep quality		√	√
	Health resources		√	√
	Quality of life		√	√
Adherence	Adherence to tailored health goals			√

**Table 3:** Details for all interventions.

Module	Content	Duration (h)
Basic modules	Welcome	1
	Physical exercise	1.5
	Healthy diet	1.5
	Social capital	1.5
	Health literacy	1.5
	Stress management	1.5
	Resilience	1.5
	→Total duration basic module	10
Core modules	Physical training	8
	Teaching kitchen	6
	Nutrition seminar	2
	Psychological support	5
	Social capital	3
	Health literacy	1.5
	Massage	1
	→Total duration core module	26.5
Elective modules	Creative workshop	3
	Individually agreed	6
	→Total duration elective modules	9
